# Pneumorrhachis, pneumomediastinum, pneumopericardium and subcutaneous emphysema as complications of bronchial asthma

**DOI:** 10.4103/1817-1737.53352

**Published:** 2009

**Authors:** Prasad K. Manden, Almas H. Siddiqui

**Affiliations:** *King Saud Chest Hospital, Riyadh, Kingdom of Saudi Arabia*

**Keywords:** Bronchial asthma, computed tomography, intra-spinal air, pneumorrhachis

## Abstract

Pneumorrhachis (PR), or epidural emphysema, denotes the presence of air in the spinal epidural space. It can be associated with a variety of etiologies, including trauma; recent iatrogenic manipulations during surgical, anesthesiological and diagnostic interventions; malignancy and its associated therapy. It usually represents an asymptomatic epiphenomenon but also can be symptomatic by itself as well as by its underlying pathology. The pathogenesis and etiology of PR are varied and can sometimes be a diagnostic challenge. As such, there are no standard guidelines for the management of symptomatic PR, and its treatment is often individualized. Frequently, multidisciplinary approach and regimes are required for its management. PR associated with bronchial asthma is extremely rare, and only very few cases are reported in the literature. Here, we report a case of a 17-year-old Saudi male patient who is a known case of bronchial asthma; he presented with extensive subcutaneous emphysema, pneumomediastinum, pneumopericardium and pneumorrhachis as complications of an acute exacerbation of his primary ailment.

The presence of air in the spinal epidural space is a rare radiological entity, mainly described in radiological literature. Various etiologies leading to the entrapment of air in the intra-spinal space, a condition called pneumorrhachis (PR), have been documented. Mainly, traumatic and iatrogenic incidents are reported in the development of this radiological epiphenomenon. PR associated with bronchial asthma is extremely rare, and only 13 cases are seen reported in the entire literature. Acute increase in the intra-alveolar pressure leads to rupture of alveoli resulting in air escape into the perivascular space. This air further moves through the facial planes into the posterior medias tinum and thus into the epidural space.

Here we present the case of a male patient who presented with an attack of bronchial asthma associated with subcutaneous emphysema, pneumomediastinum, pneumopericardium and pneumorrhachis.

## Case Report

A 17-year-old Saudi male patient was presented to our ER with history of shortness of breath, nonproductive cough and wheezing since 48 hours prior to his visit. He was a known case of bronchial asthma from early childhood, hospitalized twice in the last 2 years due to worsening of his symptoms. He was never admitted to ICU nor did he ever receive assisted mechanical ventilation in the past. Since the last 9 months, he was taking beclomethasone MDI 200 μg twice daily with p.r.n, beta-2 agonists.

There was neither any previous history of pulmonary tuberculosis or anti-tuberculous treatment nor any recent trauma or surgery. He has not been a smoker and denies any illicit drug usage.

On clinical examination, he was fully conscious and oriented, but dyspneic. He could complete a sentence at a stretch. Vitals showed PR- 104/ min, RR- 28/min, BP- 104/68 mm Hg, SPO2-89% in normal room air by pulse oxymetry and 92% with 4 L/ min oxygen delivered through nasal canula. Temperature was 38°C.

There was extensive subcutaneous emphysema involving bilateral upper chest, neck and axillary regions.

Auscultation revealed a systolic crunching sound over the left parasternal region, better heard during inspiration (Hamman' sign). The lung fields revealed bilateral extensive expiratory polyphonic wheezes with few inspiratory crackles on auscultation.

ABG on admission showed pH- 7.421, pCO_2_-31.1 mm Hg, pO_2_-54.6, SO_2_ 85.6%, HCO_3_-25.8.

**Figure 1:**
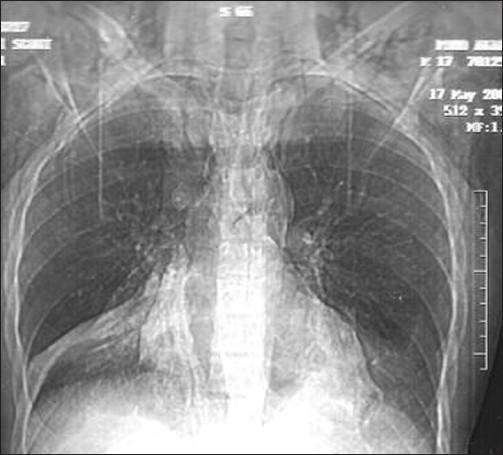
Chest x-ray showing extensive subcutaneous emphysema and pneumomediastinum

CBC: TLC- 24.3 K/μL, N- 84.4%, L- 4.7%, Hb- 17.2 gm%, Plat- 250 K/μL. All the baseline biochemical parameters were within normal limits. The initial chest x-ray revealed surgical emphysema involving the cervical, thoracic and axillary regions bilaterally without any evidence of rib fracture [Figure 1]. There was pneumonic consolidation involving the right lower lobe. It also revealed air in the mediastinum and pericardium.

**Figure 2:**
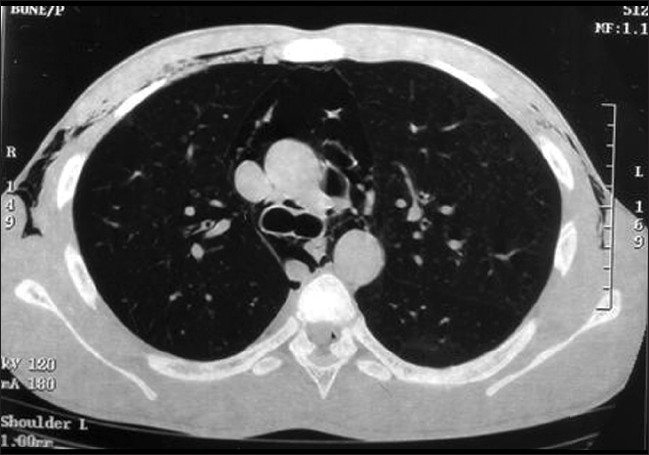
CT chest showing pneumomediastinum, pneumopericardium and surgical emphysema

**Figure 3:**
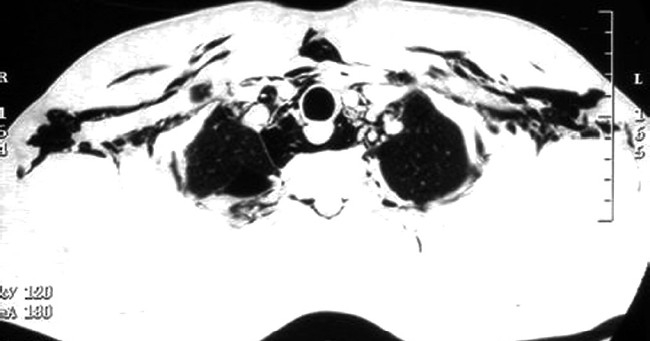
CT chest showing extensive surgical emphysema and pneumomediastinum

**Figure 4:**
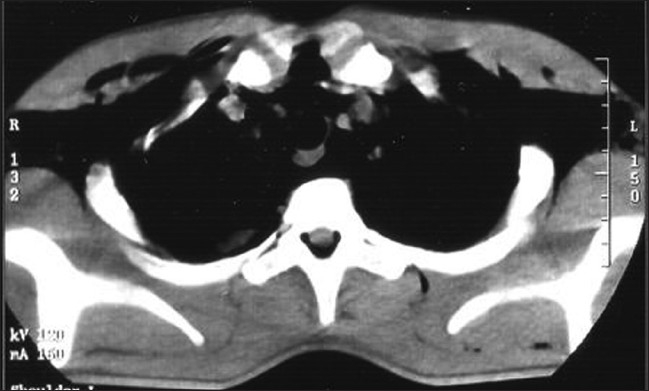
CT chest showing posterior pneumorrhachis

For better delineation of the extent of air dissection, he was subjected to computed tomography (CT) of the chest. It confirmed air dissection into the subcutaneous, mediastinal and pericardial spaces [Figures 2 and 3]. High-resolution CT also revealed air in the posterior spinal epidural space at multiple cervical and thoracic levels [Figure 4].

The patient was admitted and was vigorously treated with broad-spectrum antibiotics, inhalation bronchodilators, systemic corticosteroids, high-flow oxygen and other supportive measures. Antibiotics were used since our patient had pneumonic consolidation involving right lower lobe. His hospital course was uneventful, and he showed progressive improvement.

He was subsequently shifted to cardiothoracic care, and follow-up at 2 months revealed an asymptomatic individual.

## Discussion

PR is the presence of air in the spinal epidural space. It is an exceptional and often accidental radiological finding; it is caused by various traumatic and nontraumatic etiologies. Different pathways of air entry into the spinal canal are postulated. Only sporadic cases are reported in the literature, and no series of more than 3 cases of this condition could be found. Up to December 2005, only 71 articles were identified that reported on 86 cases. In association with bronchial asthma, only 13 cases are reported in the entire literature, caused by violent coughing and acute increase in the intra-thoracic pressure.[1]

Free air in the epidural space is an uncommon phenomenon that was first reported by Gordon *et al.* in 1977.[2] This condition was described under various terms — intra-spinal pneumocele or pneumocele; spinal, epidural and subarachnoid pneumatosis; spinal and epidural emphysema; aerorachia; pneumosaccus; air myelogram; pneumomyelography; etc., by different authors. Newbold *et al*. introduced the term pneumorrhachis in 1987.[3]

PR usually constitutes an asymptomatic, often underdiagnosed, epiphenomenon of coincident underlying injuries and diseases. With the advent of more advanced imaging techniques, more cases are being reported.

For descriptive purposes, PR can be classified into internal, intradural (intra-spinal air within the subdural or subarachnoid space), external and extradural (intra-spinal, epidural air).[1,2] External PR is usually asymptomatic and often an accidental radiological finding, whereas internal traumatic PR is often associated with fatal outcomes.[4]

Air can enter the epidural space from direct penetrating injuries of the spine. In bronchial asthma, probably, rupture of a peripheral pulmonary alveolus due to sudden increase in the intra-alveolar pressure could be the initial event. The leaked air into the pulmonary perivascular interstitium dissects the path of least resistance from the mediastinum to the facial planes of the neck. There are no facial barriers to prevent communications of the posterior mediastinum or the retro-pharyngeal space with the epidural space. Air thus freely communicates via the neural foramina and collects in the epidural space.[5] Because of the low resistance from the loose connective tissues as compared with the rich vascular network present anteriorly, the dissected air preferably collects in the posterior epidural space.[6]

Epidural PR may broadly be classified into iatrogenic, nontraumatic and traumatic.[7] Iatrogenic occurrences are the most common and result from the administration of epidural anesthesia. Nontraumatic occurrences have been described following spontaneous or non-trauma-related pneumomediastinum or pneumothorax.[5] The combination of pneumomediastinum with epidural pneumorrhachis without thoracic trauma has rarely been reported in the literature.

Almost exclusively, PR is found in combination with incidences of associated air distribution in other body cavities and facial compartments. These include pneumocephalus, pneumothorax, pneumomediastinum, pneumopericardium, subcutaneous emphysema, etc. In our patient, it was associated with the last three entities. We did not go for a CT head to diagnose a pneumocephalus.

As it is evident, PR is not a primary clinical diagnosis and is often detected accidentally. The diagnostic work-up of patients with PR should necessarily include plain roentgenograms and CT scanning of the spine.

In the differential diagnosis, intra-spinal gas collection due to degenerative,[8] malignant,[9] inflammatory[10] and infectious diseases, caused by gas-forming organisms, has to be considered. Furthermore, the coincidence of PR and intra-spinal gas also merits consideration.

Patients with pneumorrhachis are usually managed conservatively. Most of the times, PR is asymptomatic and constitutes a radiological curiosity. The intra-spinal air reabsorbs spontaneously and completely into the circulation. Rarely, symptomatic PR with features of neurological compression and consequent deficits is reported.[11]

Because of the rarity of the condition, no standard guidelines exist for the management of PR. It is thought to be associated with increased morbidity and mortality if associated with air dissection in other body cavities.[12] All the contributing factors leading to PR have to be appropriately addressed. Some mechanical deficits in the dura may warrant neuro-surgical intervention.[13,14]

Bronchoscopy for the removal of foreign body, transient high-flow oxygen therapy to facilitate nitrogen washout, etc., are employed in individual cases. The use of broad-spectrum antibiotics remains controversial,[15,16] and some schools believe that it may be beneficial in preventing possible mediastinitis; however, the decision has to be individualized. Thus, the management of patients with PR has to be decided on an individual basis and often requires inter- and multi-disciplinary approach.
